# Intraoperative use of heads-up display in skull base surgery

**DOI:** 10.3171/2021.10.FOCVID21177

**Published:** 2022-01-01

**Authors:** Laura Salgado-Lopez, Holly Oemke, Rui Feng, Stavros Matsoukas, J Mocco, Raj Shrivastava, Joshua Bederson

**Affiliations:** Department of Neurosurgery, Mount Sinai Hospital, New York, New York

**Keywords:** endoscope, heads-up display, preoperative embolization, skull base, virtual reality

## Abstract

In this video, the authors highlight the applications of virtual reality and heads-up display in skull base surgery by presenting the case of a 45-year-old woman with an incidental large clinoid meningioma extending into the posterior fossa. The patient underwent preoperative endovascular tumor embolization to facilitate tumor resection and reduce blood loss, followed by a right pterional craniotomy. The use of intraoperative Doppler, intraoperative neurophysiological monitoring, and endoscope-assisted microsurgery is also featured. A subtotal resection was planned given tumor encasement of the posterior communicating and anterior choroidal arteries. No new neurological deficits were noted after the surgical procedure.

The video can be found here: https://stream.cadmore.media/r10.3171/2021.10.FOCVID21177

We present the case of a 45-year-old woman with a right clinoid meningioma diagnosed incidentally after a head trauma. Her neurological exam was normal at the time of presentation. The tumor was extending from the parasellar and sellar regions to the middle and posterior fossae.

## 0:36 CTA.

A CTA was obtained to further characterize the intracranial arteries encased and displaced by the tumor.

## 0:42 Preoperative Angiography.

A preoperative angiogram was conducted 2 days before surgery to devascularize the lesion and reduce intraoperative blood loss.^1^ The inferolateral and meningohypophyseal trunks were coiled, resulting in reduction of tumor blush. Note the fetal origin of the right posterior cerebral artery. After the procedure the patient experienced right partial third nerve palsy.

## 1:09 Virtual Reality.

Three-dimensional simulation allows neurosurgeons in training to explore the different operative angles, as well as to plan the bone opening while using the virtual reality goggles.^2,3^ Here we can see in close detail how the tumor was encasing the internal carotid artery terminus, the origin of the anterior and middle cerebral arteries, as well as the posterior communicating and anterior choroidal arteries. The basilar and superior cerebellar arteries were in close contact with the posteromedial aspect of the tumor and laterally displaced. This 3D virtual model of the patient’s specific anatomy can also be integrated with the navigation information intraoperatively to assist during surgical resection.

## 1:51 Preoperative Planning.

The images of the CT angiography and MRI DICOM series were fused to coregister scans for preoperative planning and intraoperative navigation. Preoperative planning software allows for segmentation and 3D reconstruction of the tumor and critical neurovascular structures using the Smartbrush tool on the MRI or CT imaging. The segmented neurovascular objects can be highlighted in specific colors; here, among others, we have segmented the tumor in purple, the internal carotid and middle cerebral arteries in pink, the posterior communicating artery in blue, optic nerves in yellow, and the brainstem in green. This information can then be registered via navigation and displayed in the eyepiece of the microscope in an augmented reality fashion to optimize surgery workflow and avoid having to redirect attention to the navigation screen. The objects can be turned on and off during surgery to draw attention to specific structures intraoperatively and also limit attention blindness.^4^

## 2:57

The heads-up display has potential value during multiple stages of surgery from as early as planning the skin incision to craniotomy design or dura opening.

## 3:07 Craniotomy.

The microscope can be brought to the field to superimpose the tumor object over the patient’s anatomy and confirm appropriate size of the pterional craniotomy.

## 3:16 Dura Opening and Initial Dissection.

The dura was opened in a C-shape and reflected anteriorly. When opening the basal cisterns, special care must be taken to identify and preserve the superomedially displaced optic nerve, which can be seen outlined in yellow in the heads-up display before it becomes visible.

## 3:34

While we continue to split the sylvian fissure and advance in the subarachnoid dissection to access the tumor, we have changed the heads-up display visualization from 3D to 2D. The augmented reality can be seen as distracting and interfere with the visualization of the surgical field; however, over time and with practice the optimized number of segmented structures can easily be integrated with the anatomical visual information.^5^

## 4:01

Here we have specifically outlined the optic nerves, the internal carotid artery, the middle cerebral artery branches, and the tumor.

## 4:11 Initial Tumor Resection.

We start by carefully dissecting the tumor away from the branches of the middle cerebral artery. There is a superior and posterior offset of the heads-up display relative to the patient’s anatomy. The current software allows us to update the heads-up display information intraoperatively, but given that in this case there is just 1 or 2 mm of difference, it is not necessary to adjust it.^6^

## 4:33

Note that the solid line represents the portion of the middle cerebral artery with the current focal depth of the microscope, whereas the portions in a deeper plane are outlined in dots. By confirming that the objects displayed correspond with the patient’s anatomy, we have confidence that the information provided by the heads-up display is accurate.

After careful retraction of the frontal lobe, we visualize the lenticulostriate arteries, which limit access to the medial aspect of the tumor. Therefore, attention is taken to approach the tumor in its more anterolateral aspect.

## 5:07 Intraoperative Monitoring.

At this point, a cortical grid was placed over the motor cortex to obtain continuous monitoring from the left side of the body. Transcranial motor and somatosensory evoked potentials were also monitored bilaterally since the beginning of the surgery.

## 5:21 Anterolateral Tumor Resection.

After gaining inferior access to the most anterolateral aspect of the tumor, we dissect the tumor away from the middle cranial fossa floor. Here again, the solid line represents the portion of the tumor with the same focal depth of the microscope, whereas the tumor that is deeper to the plane of dissection is outlined in dots, providing constant awareness on how much further we have to go to continue this part of the dissection. We coagulate and dissect the tumor capsule with the bipolar before proceeding with further debulking of this portion of the tumor. The Sonopet is combined here with the bipolar to facilitate tumor debulking.

## 5:52

We can see how this part of the tumor is relatively avascular thanks to the preoperative embolization.

## 5:57 Superoposterior Tumor Resection.

The anterolateral aspect of the tumor has been debulked and now we are progressing the dissection toward its superoposterior aspect and delivering it anteriorly, into the expanded sylvian fissure. Again, we use our bipolar to dissect the tumor capsule and ultimately the Sonopet to debulk the tumor.

## 6:17 Identification of ICA and Lateral Tumor Border.

Here, we take advantage of the heads-up display to early identify the internal carotid artery on the left side of the surgical field. In addition, given that the lateral aspect of the tumor is underneath the temporal lobe, the heads-up display is also helpful to provide awareness of the lateral border of the tumor. Here we see the origin of the posterior communicating artery entering into the tumor. Just distal to the posterior communicating artery it is the origin of the anterior choroidal artery, medial to the suction tip.

## 6:48

Those arteries are protected with a cottonoid and further work is done on the tumor lateral to them.

## 6:54 Identification of PComA.

At this point, it is necessary to identify the posterior communicating and anterior choroidal arteries as they exit the tumor posteriorly. The heads-up display, here outlined in blue, is critical in order to provide situational awareness for the surgeon to identify these structures before seeing them with the microscope. This moment is one of the most important of the case, since it is essential to preserve these arteries given the crucial anatomical brain areas they supply and the fetal origin of the right posterior cerebral artery. We confirm the patency of the vessels using the Doppler.

## 7:28 Posterolateral Tumor Resection.

After confirming the vessels, just as we did anteriorly, we will now focus the remainder of the resection lateral to those vessels. Here we identify the superior cerebellar artery in the perimesencephalic cisterns medial to and beneath the tentorium.

## 7:45

This demonstrates again the efficacy of the preoperative embolization in creating relatively avascular tumor tissue. The consistency of this portion of the tumor is, however, more fibrotic than its most anterior aspect, and the use of bipolar is needed to coagulate and detach tumor pieces.

## 8:04

Here we can see the fourth nerve in the ambiens cistern just beneath the free edge of the tentorium.

## 8:10

With the superior cerebellar artery now protected underneath the cottonoids, we can see here branches of the anterior inferior cerebellar artery.

## 8:20 Integrated Navigation.

On the left, we can see how the posterior cerebral artery correlates with the integrated navigation screen, with the microscope trajectory in blue.

## 8:28 Final Tumor Resection.

By following the dissection medially and inferiorly to the posterior cerebral artery, we identify the basilar artery and dissect it from the tumor. In this final stage of the surgery, we are decompressing the brainstem by removing the last portion of the tumor in the posterior fossa accessible through this approach.

## 8:46

We can see in the heads-up display how the posterior communicating artery delineates the medial limit of our tumor resection. Endoscope-assisted microsurgery complements traditional microsurgery in the management of complex skull base tumors.

## 9:02

The ability to attach the endoscope to the microscope makes easier to combine both techniques.^7,8^ In this endoscopic view of the posterior fossa, we can see the preserved fourth nerve underneath the tentorium with some expected residual tumor.

## 9:14 Postoperative MRI.

The postoperative MRI demonstrated decompression of the brainstem with expected residual tumor medial to the posterior communicating and lenticulostriate arteries. No new neurological deficits were noted after the surgical procedure.

## Disclosures

Dr. Mocco reported nonfinancial support from Penumbra, EndoStream, Stryker, and MicroVention outside the submitted work.

## Author Contributions

Primary surgeon: Bederson. Assistant surgeon: Salgado-Lopez, Mocco. Editing and drafting the video and abstract: Salgado-Lopez, Oemke, Feng, Matsoukas, Bederson. Critically revising the work: Salgado-Lopez, Oemke, Feng, Matsoukas, Mocco, Shrivastava, Bederson. Reviewed submitted version of the work: Salgado-Lopez, Oemke, Feng, Mocco, Shrivastava, Bederson. Approved the final version of the work on behalf of all authors: Salgado-Lopez. Supervision: Salgado-Lopez, Oemke, Mocco, Shrivastava, Bederson.
